# Experimental and simulation study on aluminium alloy piston based on thermal barrier coating

**DOI:** 10.1038/s41598-022-15031-x

**Published:** 2022-06-29

**Authors:** Yang Liu, Jilin Lei, Xiaoqiang Niu, Xiwen Deng, Jun Wen, Zhigao Wen

**Affiliations:** 1 Yunnan Key Laboratory of Internal Combustion Engines, Kunming University of Science and Technology, Kunming, 650500 People’s Republic of China; 2 Yunnan Key Laboratory of Plateau Emission of Internal Combustion Engines, Kunming Yunnei Power Co., Ltd, Kunming, 650200 People’s Republic of China; 3 Chengdu Galaxy Power Co., Ltd, Chengdu, 610505 People’s Republic of China

**Keywords:** Engineering, Mechanical engineering

## Abstract

Thermal barrier coatings (TBCs) have low thermal conductivity, effectively reducing the temperature of the metal matrix and improving thermal performance, knock resistance, and combustion performance of the piston. In this study, an off-road high-pressure common-rail diesel engine was chosen as the research object. Combined with the test results of the piston temperature field under the rated power and maximum torque conditions, a finite element simulation model of the thermal barrier coating piston was established. This model enabled the distribution characteristics and variation laws of the temperature field, stress, and deformation of the thermal barrier coating on the piston matrix to be analysed. The results show that the maximum temperature of the TBC piston is 12.2% and 13.73% lower than that of the aluminium alloy piston under the rated power and maximum torque conditions, respectively. The thermal stresses of the TBC piston at the top of the cavity were 25.9% and 26.8% lower than those of the aluminium piston, while the thermo-mechanical coupling stress of the TBC piston was slightly higher than that of the aluminium piston—1.2 MPa and 3.7 MPa in the bottom of the combustion chamber with geometric mutation, respectively. The radial thermal deformation of the TBC piston was 0.067 mm and 0.073 mm lower than that of the aluminium piston, with the radial thermo-mechanical coupling deformation also decreasing by 0.069 mm and 0.075 mm, respectively. The radial thermal deformation of the piston in the direction parallel to the pinhole axis was greater than that in the direction perpendicular to the pinhole axis; the difference in the magnitude of the change results in uneven thermal deformation of the piston.

## Introduction

Diesel engines are continuously being developed to improve their durability, lightweightedness, compactness, and low emission capabilities. As a result, the rate of heat released and the temperature and pressure within the cylinder has significantly increased, worsening the environment within the cylinder^1–4^. The piston, the core component within a diesel engine cylinder, is subjected to increasing thermoshock, significantly affecting its reliability and durability. Thermal barrier coatings (TBCs) provide excellent thermal insulation and are beneficial for anti-erosion, thermoshock resistance, and high-temperature oxidation resistance^5,6^. In engineering practices, these characteristics can significantly affect the heat transfer between the working substrate and the high-temperature environment, thereby affecting the structural strength and fatigue life of the working substrate^7,8^. The TBC on the top of the piston can effectively reduce the temperature at the top of the piston and significantly improve the combustion and thermal performances, and engine resistance to knock^9^.

With the rapid development of TBC preparation technology, research on pistons with TBCs has been conducted with several studies investigating the influence of TBCs on the performance of internal combustion engines. As early as the 1980s, Morel et al.^10,11^ conducted a numerical model assessment of the piston and head of a heavy-duty diesel engine. The piston was coated with a 1.5 mm zirconia plasma spraying (ZPS) coating. The thermal efficiency of the engine was found to increase by approximately 5%. Taymaz^12^ showed that TBCs improve the thermal efficiency of diesel engines, thus reducing fuel consumption at different speeds and loads. Toyota proposed a new heat insulation concept in a combustion chamber known as Thermo-Swing Wall Insulation Technology (TSWIN). A novel insulation material—silica reinforced porous anodized aluminium (SiRPA), with low thermal conductivity and low volumetric heat capacity—as developed and applied it to the top surface of the piston, resulting in reduced heat loss without sacrificing engine performance, thereby improving the thermal efficiency^13–16^.

Moreover, the effects of pistons with TBCs on engine pollutant emissions have been investigated in several studies. Ciniviz et al.^17^ investigated the TBC effects on the top of a piston and combustion chamber surface and on the emission performance of turbocharged diesel engines. The results showed that NOx emissions increased by 10% while soot emissions decreased by 18%, compared with standard diesel engines. Cerit et al.^18^ found that the TBC on a piston can reduce the HC emissions of a gasoline engine cold start by 43.2%, without any degradation in the engine performance, through a single-cylinder gasoline engine test study. Durat^19^ investigated the effect of TBCs, formed by adding different stabilisers to partially stabilised zirconia, on the HC emissions of gasoline engines under cold start and steady state conditions using the finite element method. The results showed that Y_2_O_3_ was more effective than MgO. Reddy et al.^20^ showed that the CO and HC emissions of coated piston engines were reduced by 16.1% and 22.5%, respectively, while NOx emissions were increased by 17.7%, compared with standard pistons.

Researchers also found that the roughness and porosity of the TBC had a significant impact on the engine combustion process and engine efficiency^21–25^. Serrano et al.^21^ discovered a 3% reduction in engine efficiency using piston TBCs. Studies by Uchida and Osada^22^ have shown that surface roughness and porous structure are the main causes of thermal boundary layer thinning in zirconia coatings, resulting in increased heat transfer coefficients. Caputo et al.^25^ showed experimentally that the roughness of the coating reduced both the rate of the combustion process and the engine efficiency (up to 2% at lower load and speed).

Additionally, several studies have investigated the TBC piston itself. Chen et al.^26^ used a plasma spraying method to apply a 0.33 mm thick zirconia ceramic layer on the top surface of an aluminium piston. The study found that the average temperature at the first ring groove of the piston decreased by 12 °C. Szymczyk^27^ showed that in certain areas, the calculated temperature of coated piston surface was approximately 40% lower than that of the uncoated piston. Buyukkaya et al.^28^ showed that the temperatures of the top of an aluminium alloy piston and a steel piston, applied with a TBC, decreased by 48% and 35%, respectively. Hejwowski et al.^29^ showed that flame-sprayed coatings are more easily damaged than plasma sprayed coatings, largely due to the formation of oxides at the bonding surface of the coating and the decomposition of Al_2_O_3_-40%TiO_2_. Feng^30^ used a simulated cylindrical piston to study the effect of a functionally graded coating on the thermal load on the top of the piston. A functionally graded coating can increase the temperature of the combustion chamber and improve the strength, wear resistance, and sealing properties of the piston.

The research on TBC pistons has mainly focused on the influence of the TBC on the temperature field of the piston matrix, engine efficiency, combustion process, and emission performance. However, few studies have focused on the thermal analysis and thermo-mechanical coupling of the TBC piston system, while numerical simulation analysis of the TBC piston has often not been experimentally verified. Therefore, using an aluminium alloy piston of an off-road diesel engine as the research object, a finite element simulation analysis model of the TBC piston is established by combining the temperature test and the maximum combustion pressure in the cylinder. The purpose of this study is to investigate the effect of the TBC on the temperature field, stress, and deformation of the piston matrix. The results of this study can also provide data support and reference for optimising the kinematic and dynamic performance and improving the cylinder clearance of pistons.

## Methods

### Research subject

The research subject is an off-road high-pressure common-rail diesel engine, Wherein the aluminium alloy piston is cooled by an internal cooling gallery and the piston chamber shape is an indented ω type. The relevant parameters for the diesel engine are listed in Table 1.

**Table 1:** Main parameters of the engine.

Parameter	Value
Engine displacement (L)	3.92
Calibrated power (kW)	125
Calibrated speed (r/min)	2600
Maximum torque (N m)	600
Maximum torque speed (r/min)	1300–1900
Compression ratio	17.2
Bore × stroke (mm)	102 × 120

### Experiment program

The piston is located within the engine, surrounded by the cylinder liner and body. The piston head is impacted by high- temperature gas and is in a state of high-speed reciprocating motion. Therefore, the accurate measurement of the steady-state temperature at the top of the piston head has been challenging while studying steady-state thermal loads. In this study, a TT-K-30 thermocouple and lead transmission system were used to measure the temperature field of the diesel engine piston under the rated power and maximum torque conditions. The test temperature range of the thermocouple was 0–1250 °C with a tolerance of 1.1 °C or 0.4%. The thermocouple utilized OMEGA non-impurity solder joint technology to weld into spherical solder joints, thus ensuring the accuracy of measurements. Additionally, the bare type can improve the response speed, and the response time was less than 5 ms. Before the piston temperature field test, the thermocouple used was pre-calibrated from 0 to 400 °C. The technical parameters of the thermocouple sensor are listed in Table 2.

**Table 2:** Technical parameters of the thermocouple sensor.

Parameter	Value
Output voltage (V)	0–4
Calibration range (°C)	0–+400
Sensitivity (mV/°C)	10
Thermocouple response time (ms)	≤ 5
Output voltage error (%)	≤ ± 0.4
Working environment temperature (°C)	0–35
Working environment humidity (%RH)	30–90
Preheating time (min)	≤ 15
Additional error of ambient temperature (°C)	≤ ± 0.3
Continuous working time of the instrument (h)	4

**Figure 1:**
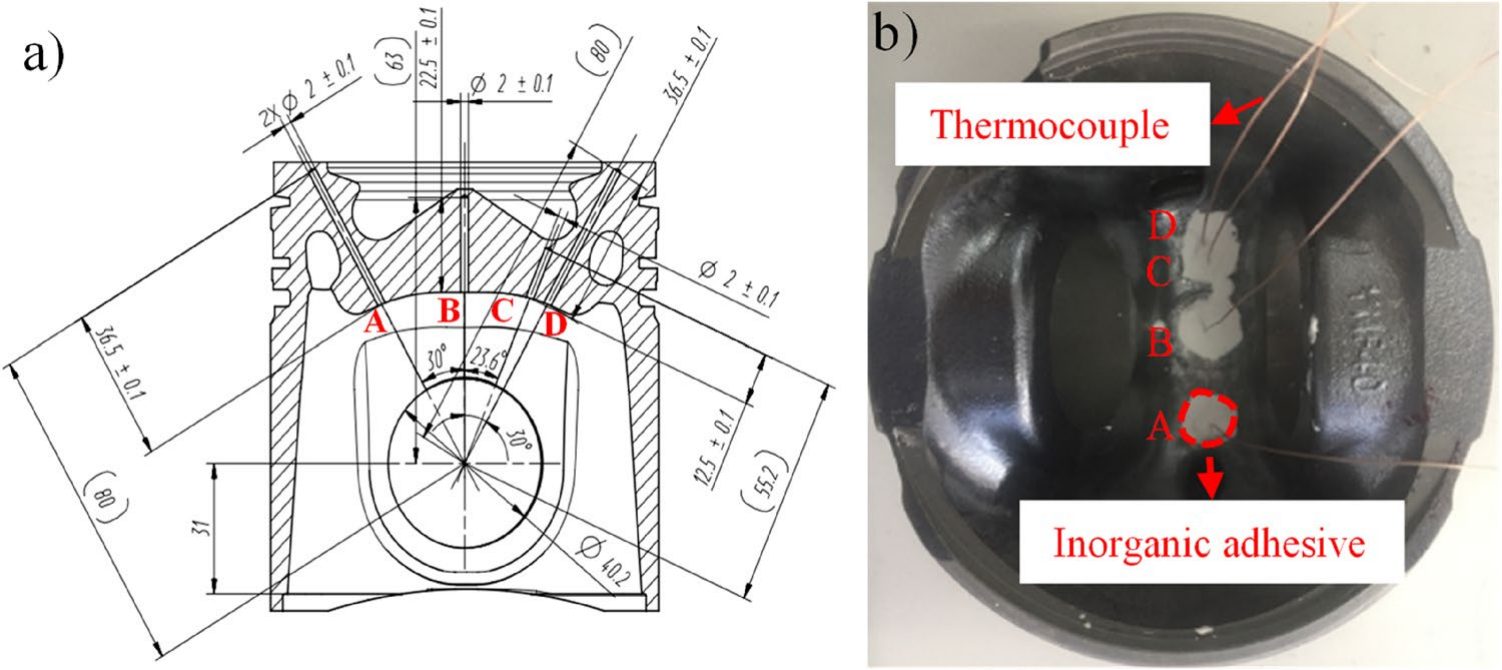
Sketch map of piston measuring points of (**a**) cutaway view, and (**b**) physical view.

A single piston was arranged with four measurement points located at the centre of the piston combustion chamber, bottom of the piston chamber, and top surface of the piston. According to the position of the measuring point, a hole was drilled from the inner cavity of the piston to a position 2 mm below the top surface of the piston. The measuring point of the thermocouple was placed into the bottom of the hole and the copper oxide inorganic glue was poured to secure, as shown in Fig. 1. During the drilling process, the drilling angle and depth should be monitored closely to avoid deviations in the drilling position, and the internal cooling gallery should be penetrated. After the thermocouple sensors were installed, the thermocouples were numbered to facilitate recording of the measurement results to ensure the integrity of the sensor using a voltmeter.

**Figure 2:**
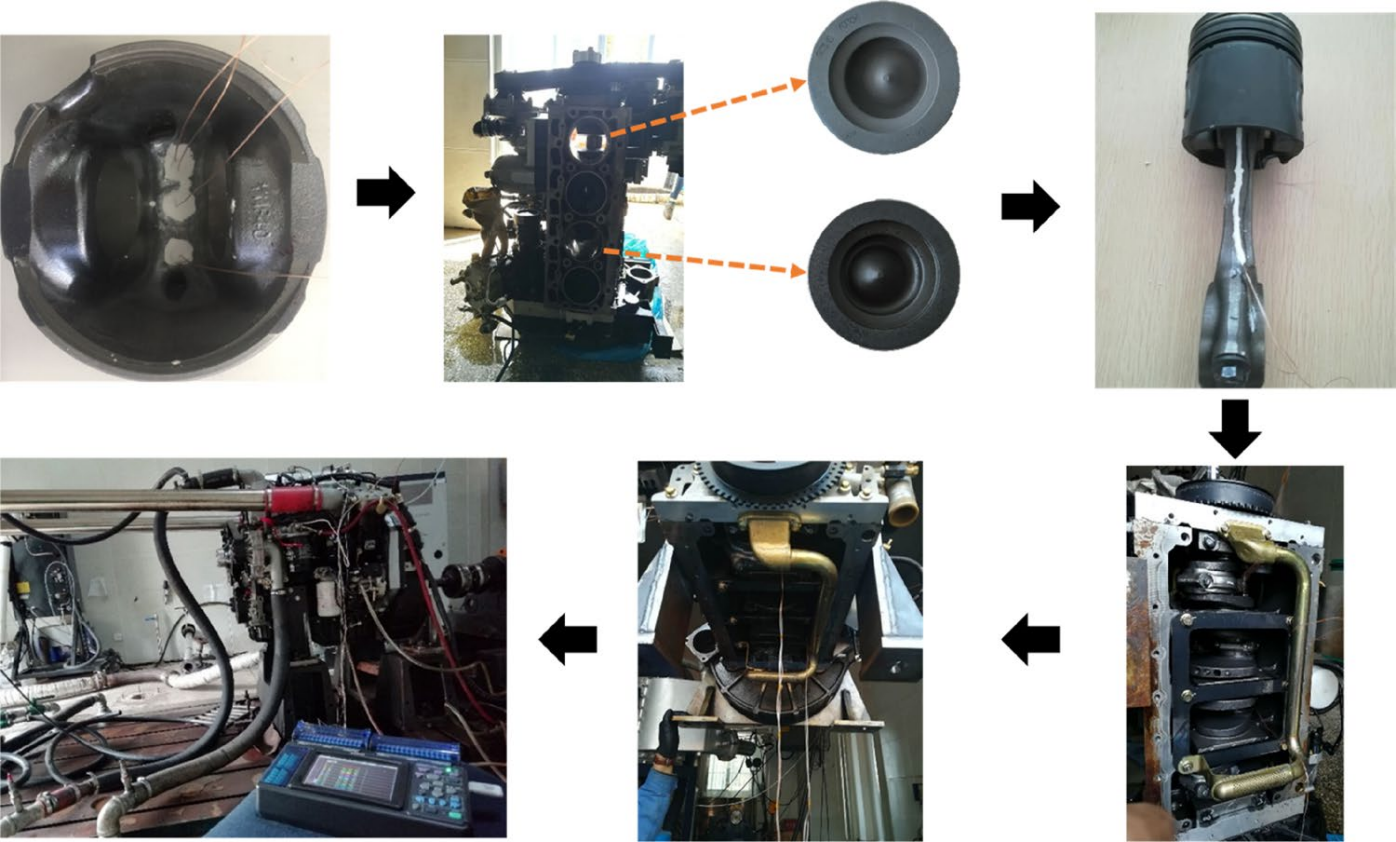
Lead physical diagram of thermocouple wire.

Considering the process of the short test, no auxiliary mechanism was introduced in the lead process; only slots were made on either side of the connecting rod, as shown in Fig. 2. The thermocouple wire was placed in the connecting rod groove, and poured the copper oxide inorganic glue to secure.

During the test, the aluminium alloy piston was installed in the first cylinder, and the TBC piston was installed in the third cylinder to evaluate the temperature field under the rated power and maximum torque conditions. The test and test conditions are shown in Table 3.

**Table 3:** Test and test conditions.

Scheme	Test condition
A	Aluminium alloy piston under the rated power condition
B	Aluminium alloy piston under the maximum torque condition
C	TBC piston under the rated power condition
D	TBC piston under the maximum torque condition

Generally, the TBC structure has a ceramic layer with high melting point, chemical stability, phase stability, low thermal conductivity, low thermal capacity, good thermo-mechanical properties, good compatibility with the metal bonding layer, low sintering rate, toughness, hardness, and good erosion wear resistance. This layer functions as heat insulation that reduces the temperature of piston matrix. The metal bonding layer, made of metal cobalt-based (CoNiCrAlY) or nickel-based (NiCoCrAlY) superalloys, is thinner than the ceramic layer^25^. As the transition layer between the piston base and ceramic layer, the metal bonding layer is used to improve the bonding strength between the ceramic layer and piston base and to reduce stress caused by the thermal expansion mismatch. Currently, zirconia (ZrO_2_) is the most widely used ceramic layer material in automotive research^25^. In this study, MgZrO_3_, with a MgO stabilizer, was used as the ceramic layer material, with high melting point, low thermal conductivity, and good stability at elevated temperatures. NiCrAl, which can enhance the bonding force and oxidation resistance of the ceramic layer to the substrate, was used as the bonding layer material of the TBC on the top surface of the piston. To alleviate the thermal expansion mismatch between the ceramic layer and the piston substrate, the metal bonding layer of the TBC on the top of the piston is generally 0.15 mm thick^12,19,25^. Previous studies^29^ have shown that if the total thickness of the TBC is over 0.5 mm, the adhesion of the TBC will decrease, affecting the volumetric efficiency of diesel engines. The greater the coating thickness, the higher the temperature, thus carbonising the lubricating oil, resulting in carbon deposition and other problems. If the total thickness of the thermal barrier coating is less than 0.5 mm, the improvement in thermal efficiency is minor, and the insulation effect is limited. Therefore, plasma arc spraying technology was used to spray ceramic layers with a thickness of 0.35 mm and metal bonding layers with a thickness 0.15 mm on the top surface of the piston.

### Simulation model setup

This chapter involved.

### Finite element model

**Figure 3:**
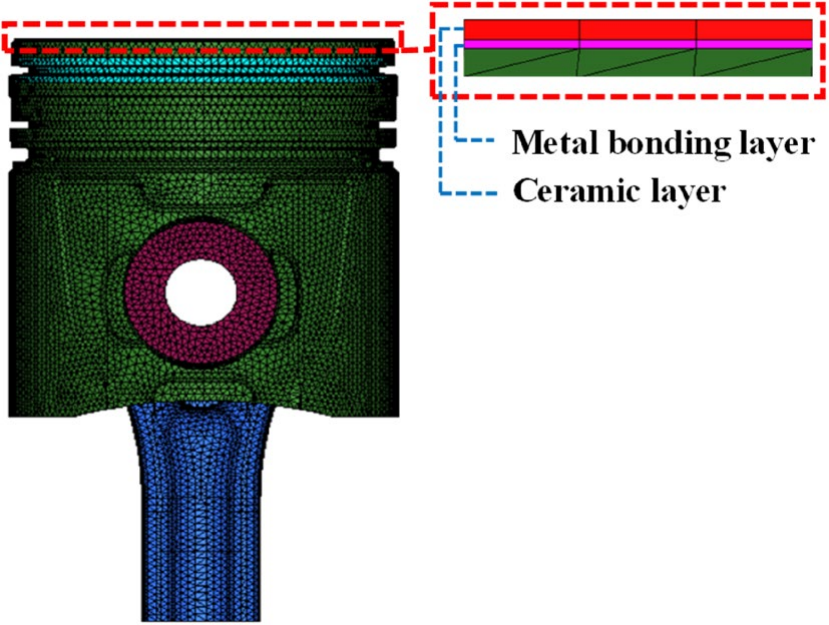
Piston finite element model and amplification region.

In this study, the HYPERMESH software was used to divide the finite element mesh of the model, and the ABAQUS software was used to build a finite element simulation model of the piston. The model comprised a piston body, ring, pin, half of the connecting rod, bonding layer, and ceramic layer, as shown in Fig. 3.

### Material characteristics parameters

The piston body material was a silicon aluminium alloy (with a small amount of magnesium), and the piston ring material was austenitic, wear-resistant cast iron. The thermophysical parameters of the silicon aluminium alloy materials at different temperatures are listed in Table 4. The specific thermophysical parameters of the piston ring, pin, connecting rod, bonding layer, and ceramic layer are listed in Table 5.

**Table 4:** Thermophysical parameters of the piston body material at different temperatures.

Temperature, ℃	Thermal conductivity, W m^−2^ K^−1^	Density, kg m^−3^	Specific heat capacity, J kg^−1^ K^−1^	Linear expansion rate, 10^−6^ m m^−1^ K^−1^
20	130	2810	900	19.2
100	134	2805	920	19.2
150	136	2798	942	19.8
200	139	2791	968	20.5
250	142	2785	973	20.8
300	143	2777	985	21.1
350	145	2773	992	21.3
400	146	2767	1007	21.5

**Table 5:** Thermophysical parameters of the piston ring, pin, connecting rod, metal bonding layer, and ceramic layer.

part	Thermal conductivity, W m^−2^ K^−1^	Density, kg m^−3^	Specific heat capacity, J kg^−1^ K^−1^	Linear expansion rate, 10^−6^ m m^−1^ K^−1^
Ring	44	7300	470	1.80
Pin	50.66	7830	510	1.37
Rod	50.66	7830	510	13.65
NiCrAl	16.1	7870	764	12
MgZrO_3_	0.8	5600	650	8

### Boundary conditions

**Figure 4:**
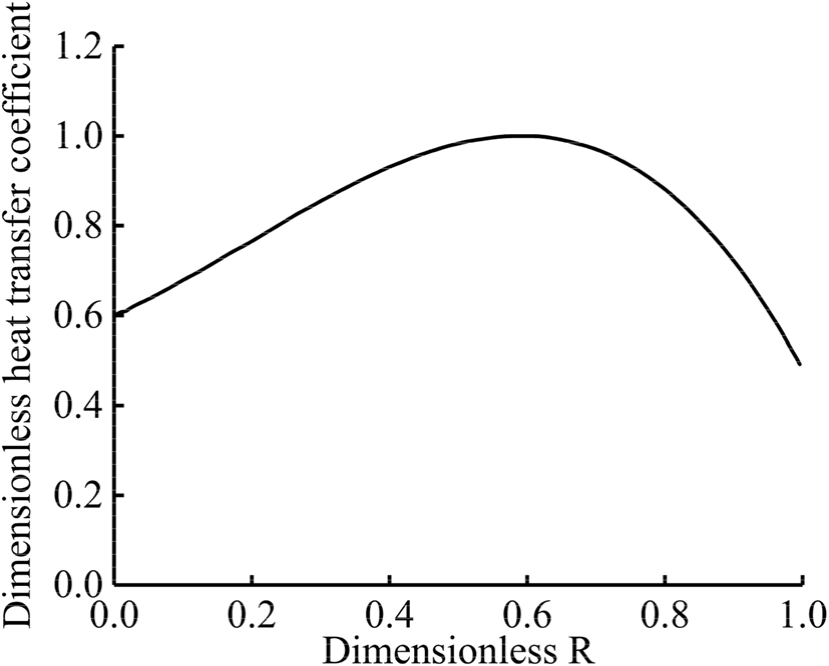
Dimensionless curve of convective heat transfer coefficient with radial distance.

Accurate thermal boundary conditions are the basis for studying the temperature field and thermal load of the piston, the key factors that determine the accuracy of the model's calculations. In this study, boundary conditions of the third type were used. The results calculated using the empirical formula were compared with the test results and repeatedly corrected to obtain accurate thermal boundary conditions. Based on the test data of the diesel engine, a one-dimensional thermodynamic simulation model was established to obtain the temperature and convective heat transfer coefficient of the gas in the cylinder under the rated power and maximum torque conditions. Considering factors such as the change in the air flow in the cylinder and the structure of the cylinder, the dimensionless relationship curve of the convective heat transfer coefficient with radial distance at the location of the piston combustion surface was calculated, as shown in Fig. 4. The ordinate is the ratio of the heat transfer coefficient of the top surface of the piston, along the radial direction at different positions, to the maximum convective heat transfer coefficient in the cylinder. The abscissa is the ratio of the top surface of the piston, along the radial direction at different positions, to the piston radius.

**Figure 5:**
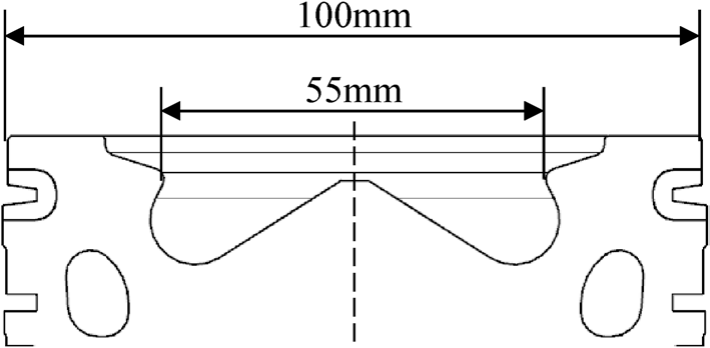
Dimensions of the top surface of the piston.

**Figure 6:**
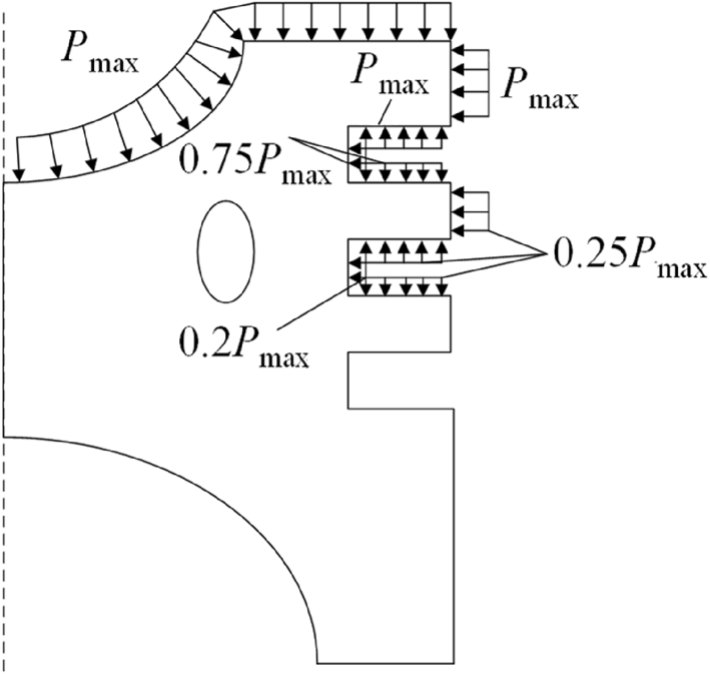
Gas pressure of the piston.

Thus, the heat transfer coefficient at any position in the radial direction of the piston combustion surface is obtained, as show in Eq. (1).

1 $$ \alpha_{r} = \left\{ \begin{gathered} \frac{{2 \cdot \alpha_{gm} }}{{1 + e^{{0.1 \cdot \left( {\frac{N}{25.4}} \right)^{1.5} }} }}e^{{0.1 \cdot \left( {\frac{r}{25.4}} \right)^{1.5} }} \;\;,{\kern 1pt} {\kern 1pt} 0 < r \le N \hfill \\ \frac{{2 \cdot \alpha_{gm} }}{{1 + e^{{0.1 \cdot \left( {\frac{N}{25.4}} \right)^{1.5} }} }}e^{{0.1 \cdot \left( {\frac{2N - r}{{25.4}}} \right)^{1.5} }} {\kern 1pt} {\kern 1pt} ,{\kern 1pt} {\kern 1pt} {\kern 1pt} r > N \hfill \\ \end{gathered} \right. $$

where *a*_*gm*_ denotes the average convective heat transfer coefficient of the gas in the cylinder. *N* is the distance from the central axis of the piston to the position of the maximum heat transfer coefficient (i.e., the throat). The position of the throat is 27.5 mm away from the central axis of the piston (hence, *N* = 27.5 mm), as shown in Fig. 5. The maximum combustion pressure of the diesel engine was 16 MPa, indicating that the gas pressure on the top of the piston was 16 MPa. As the gas pressure in the top land of the piston, first ring groove, second land, and second ring groove cannot be measured, pressures are distributed empirically by different percentages of the gas pressure of the top surface, as shown in Fig. 6. The area between the third land and skirt is subjected to less gas pressure and is therefore negligible, and this part is not subjected to any mechanical load.

### Simulation model validation

**Figure 7:**
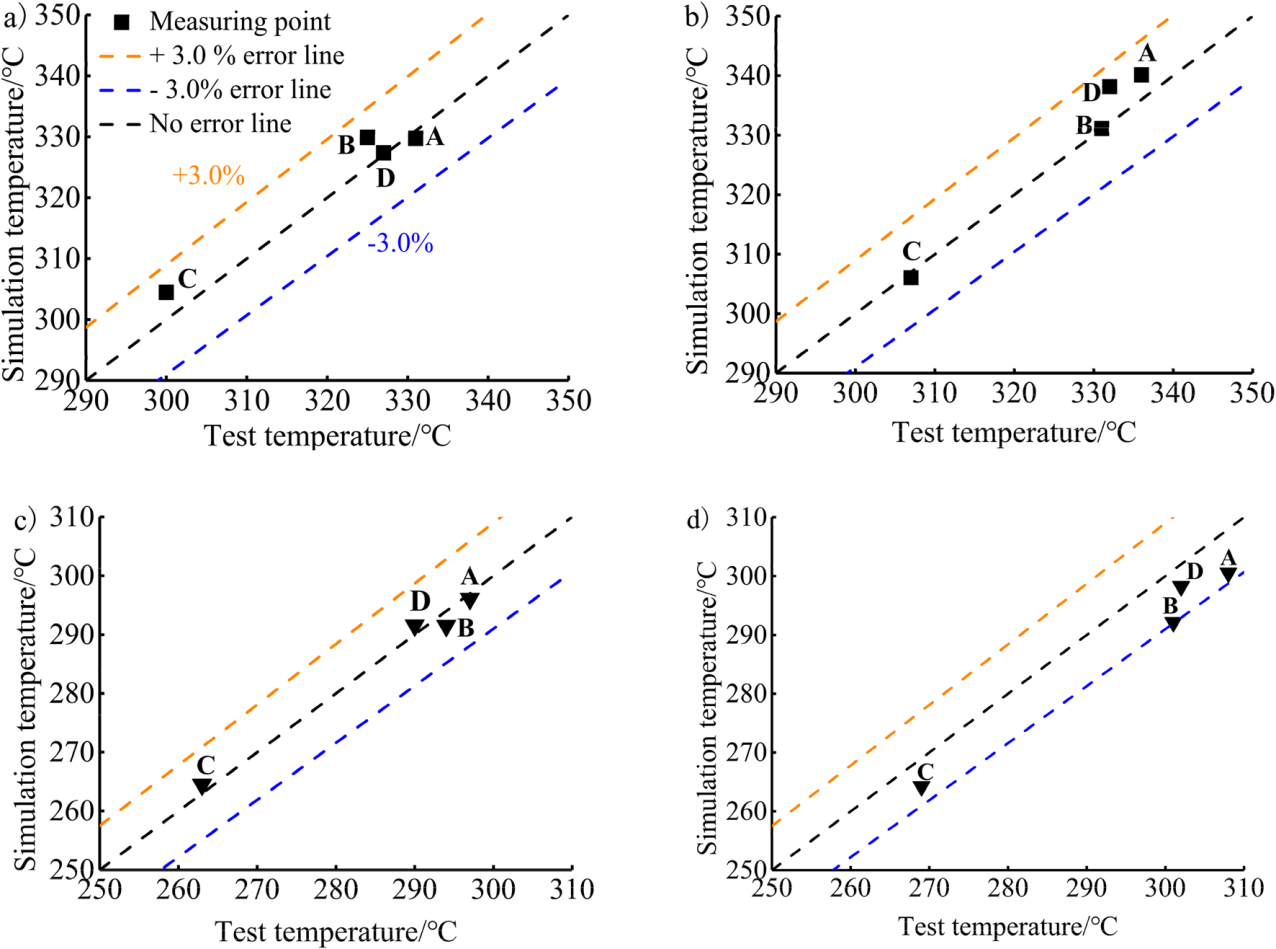
Comparison of experimental test and simulation temperatures of the piston of (**a**) Scheme A, (**b**) Scheme B, (**c**) Scheme C, and (**d**) Scheme D.

The four simulation values of the aluminium alloy piston and TBC piston simulation model were extracted and compared with the test values, as shown in Fig. 7. The results show that the percentage deviation between the temperature calculated by the simulation and the temperature of experimental test is less than 3.0%, indicating that the simulation results have high accuracy and can be used for subsequent analyses and research.

## Results and discussions

### Influence of thermal barrier coating on piston temperature field distribution

**Figure 8:**
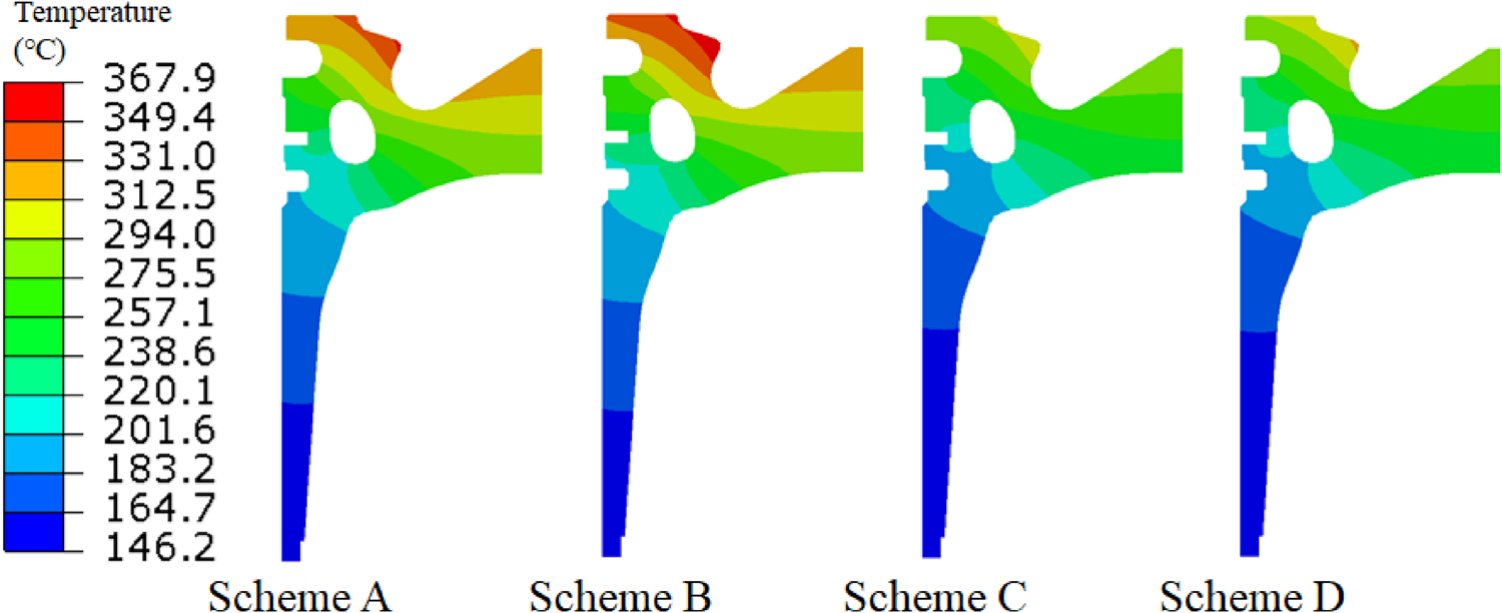
Temperature field distribution of piston.

The temperature field distributions of the different schemes were obtained via simulations, as shown in Fig. 8. The maximum temperature of the piston matrix of each scheme occurred at the throat of the piston. The maximum temperature of TBC piston in the rated power and maximum torque conditions were 310.7 °C and 317.4 °C, respectively, 43.3 °C and 50.5 °C lower than the similar maximum temperatures of the aluminium alloy piston of 354.0 °C and 367.9 °C, respectively, representing decreases of 12.2% and 13.73%, respectively. These results show that the TBC can effectively block the heat transfer from the combustion chamber to the head of the piston, significantly reducing the temperature of the h piston head. Since the area below the second land of the piston is far away from the TBC, it is less affected by the TBC, and the heat transfer coefficient of this area does not changed (i.e., the temperature difference is relatively small).

**Figure 9:**
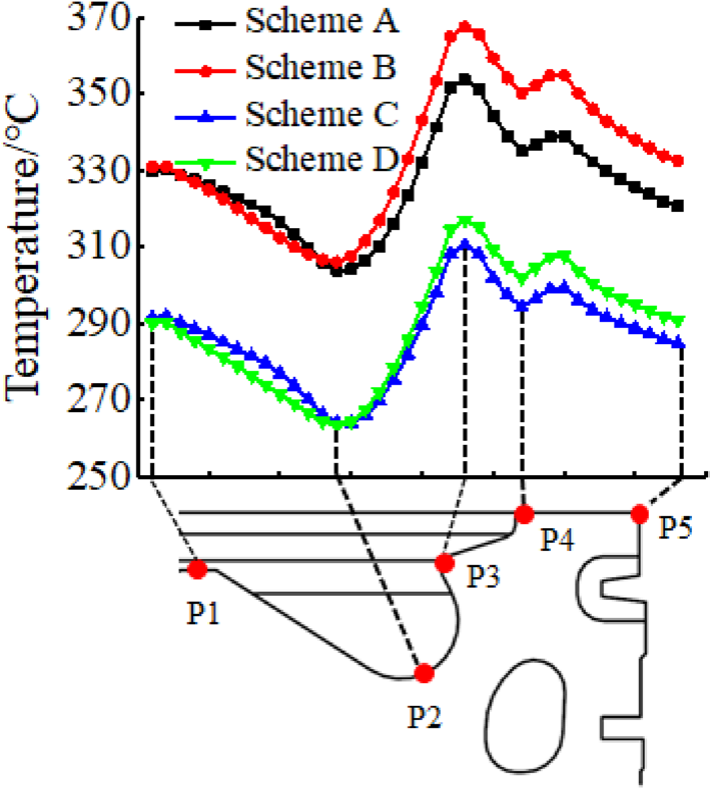
Radial temperature distribution on the top surface of the piston.

The top surface temperature of the piston in each scheme is regularly distributed along the radial direction of the piston. To better display the radial distribution law of the piston temperature, paths P1 to P5 are taken at the top surface of the piston matrix, perpendicular to the direction of the pinhole, as shown in Fig. 9. The temperature of the convex area in the centre of the piston chamber was relatively high. With an increase in the radial distance and the influence of the geometric structure, the temperature of the combustion chamber gradually decreased. The minimum temperature was reached on the bottom surface of the combustion chamber and the piston temperature then increased along the radial distance. Subsequently, the piston temperature increased along the radial distance and reached the maximum temperature at the position P3 of the throat. Further increase in radial distance resulted in a gradual decrease in the piston temperature. However, at position P4, where the geometric structure of the piston suddenly changes, the piston temperature increases dramatically, and then continues to decrease until the piston edge.

When the piston matrix temperature changes, its volume will undergo changes, which may be subject to external or mutual constraints between its various parts, resulting in thermal stress and thermal deformation. The thermal–mechanical coupling stress and deformation are the result of mechanical load bearing based on the temperature field. The effects of barrier coatings on the stress and deformation distributions are discussed below.

### Influence of thermal barrier coating on piston stress distribution

**Figure 10:**
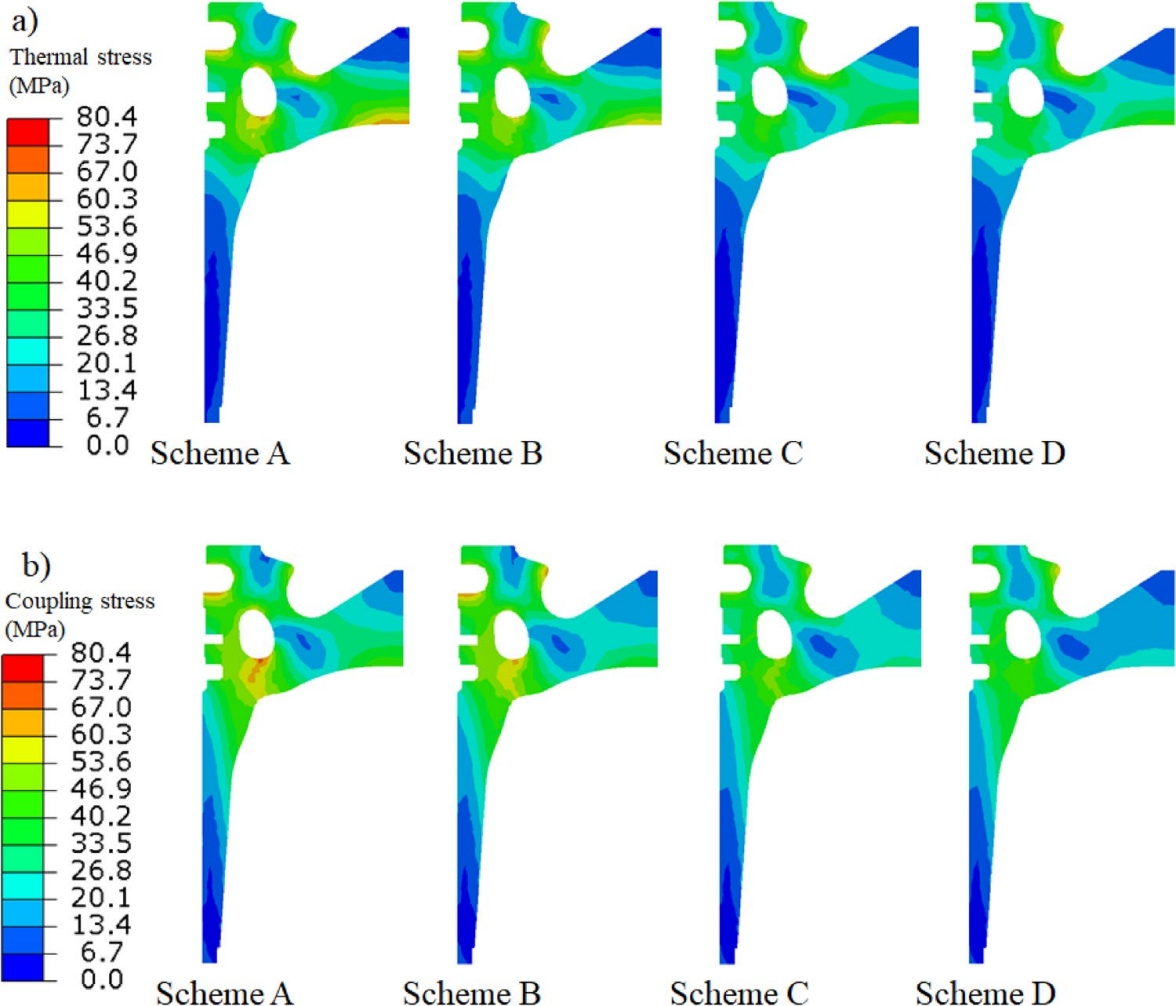
Distribution results of (**a**) thermal stress of the piston and (**b**) thermo-mechanical coupling stress of the piston.

The stress distribution of the different schemes was obtained from simulation calculations, as shown in Fig. 10. The thermal stress at the key position of the piston was compared with the thermo-mechanical coupling stress, as listed in Table 6. The thermal insulation function of the thermal barrier coating reduces the temperature gradient of the piston such that the thermal stress at the bottom surface of the combustion chamber, throat, first ring groove, bottom of the cooling gallery, and top of the inner cavity of the TBC piston is significantly lower than that of the aluminium alloy piston. Under the rated power and maximum torque working conditions, the TBC piston has the largest drop of 25.9% and 26.8% at the top of the inner cavity compared with the aluminium alloy piston. The thermo-mechanical coupling stress of the TBC piston and aluminium alloy piston is mainly reflected in the throat and first ring groove compared with the thermal stress. The thermo-mechanical coupling stress at the throat of Scheme A, B, C and D increased by 11.7%, 10.3%, 7.5% and 7.6%, respectively, compared to thermal stress; similarly the first ring groove increased by 3.2%, 3.0%, 2.4% and 2.4%, respectively. The restraining effect of the thermal barrier coating on the top surface results in the TBC piston generating slightly higher thermo-mechanical coupling stresses. Stress increases of 1.2 MPa and 3.7 MPa, compared to the aluminium alloy piston, under the rated power and maximum torque conditions, respectively, were observed at the bottom surface of the combustion chamber.

**Table 6:** Comparison of thermal stress and coupling stress at key positions of the piston.

Position	Thermal stress of the scheme (MPa)				Thermo-mechanical coupling stress of the scheme (MPa)			
	A	B	C	D	A	B	C	D
Bottom surface of combustion chamber	61.7	52.1	58.5	51.8	41.4	33.2	42.6	36.9
Throat	48.6	59.1	47.8	53.8	54.3	65.2	51.4	57.9
First ring groove	69.1	70.4	53.9	53.9	71.3	72.5	55.2	55.2
Bottom of the cooling gallery	46.0	43.7	34.5	32.4	45.6	43.7	35.5	33.9
Top of the inner cavity	64.4	57.9	47.7	42.4	44.5	38.1	28.9	23.9

### Influence of thermal barrier coating on piston deformation distribution

**Figure 11:**
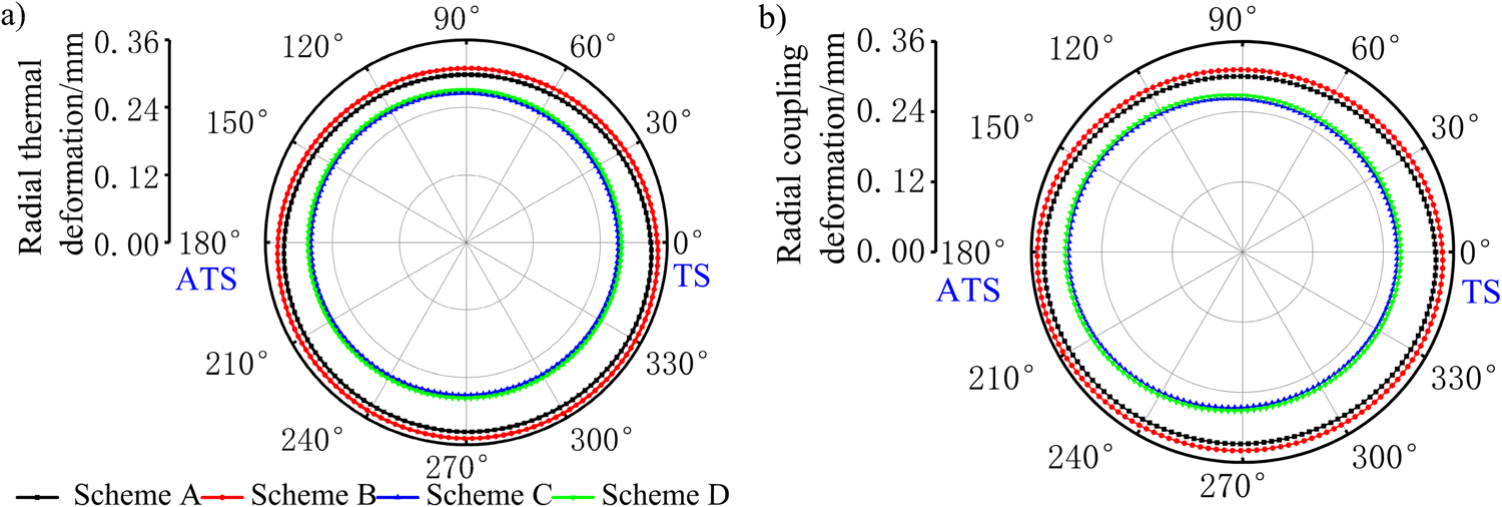
Results of (**a**) thermal deformation at the top of the piston and (**b**) thermo-mechanical coupling deformation at the top of the piston.

The deformation of the piston directly affects the fit clearance between the piston and cylinder liner during the reciprocating motion of the piston. Clearance is an important parameter that affects the kinematic and dynamic performance of the piston. Considering these factors, the polar diagram of the radial thermal deformation and radial thermo-mechanical coupling deformation at the top of the piston is shown, where TS and ATS represent the main and secondary thrust sides of the piston, respectively, as shown in Fig. 11. As seen in Fig. 11, a piston pin offset of 0.5 mm causes the radial deformation of the piston in each scheme, along the pin axis, to be greater than the deformation perpendicular to the pin axis. The radial deformation of piston under the maximum torque condition was larger than that under the rated power condition. The thermal barrier coating is closely combined with the top surface of the piston matrix, which limits the radial deformation of the top surface. The radial thermal deformation of the TBC piston, under the rated power and maximum torque working conditions, had a maximum reduction of 0.067 mm and 0.073 mm, respectively. Similarly, the radial thermo-mechanical coupling deformation had a maximum reduction of 0.069 mm and 0.075 mm, respectively, compared with the aluminium alloy piston. The minimum radial thermal deformation of the aluminium alloy piston was 0.298 mm and 0.309 mm, respectively, and appeared in the direction of 92.9°. The minimum radial thermal deformation of the TBC piston was 0.266 mm and 0.271 mm, respectively, and appeared in the direction of 76.6°. The maximum radial thermal deformation of the aluminium alloy piston was 0.338 mm and 0.350 mm, respectively, and appeared in the direction of 304.3°, while the maximum radial thermal deformation of the TBC piston was 0.278 mm and 0.283 mm, respectively, and appeared in the direction of 190.5°. The minimum radial thermomechanical coupling deformation of the aluminium alloy piston was 0.300 mm and 0.311 mm, respectively, and appeared in the direction of 82.5°. The minimum radial thermomechanical coupling deformation of the TBC piston was 0.258 mm and 0.263 mm, respectively, and appears in the direction of 60.4°. The maximum radial thermo-mechanical coupling deformation of the aluminium alloy piston was 0.340 mm and 0.351 mm, respectively, and appeared in the direction of 197.4°. The maximum radial thermo-mechanical coupling deformation of the TBC piston was 0.297 mm and 0.302 mm, respectively, and appeared in the direction of 178.8°.

**Figure 12:**
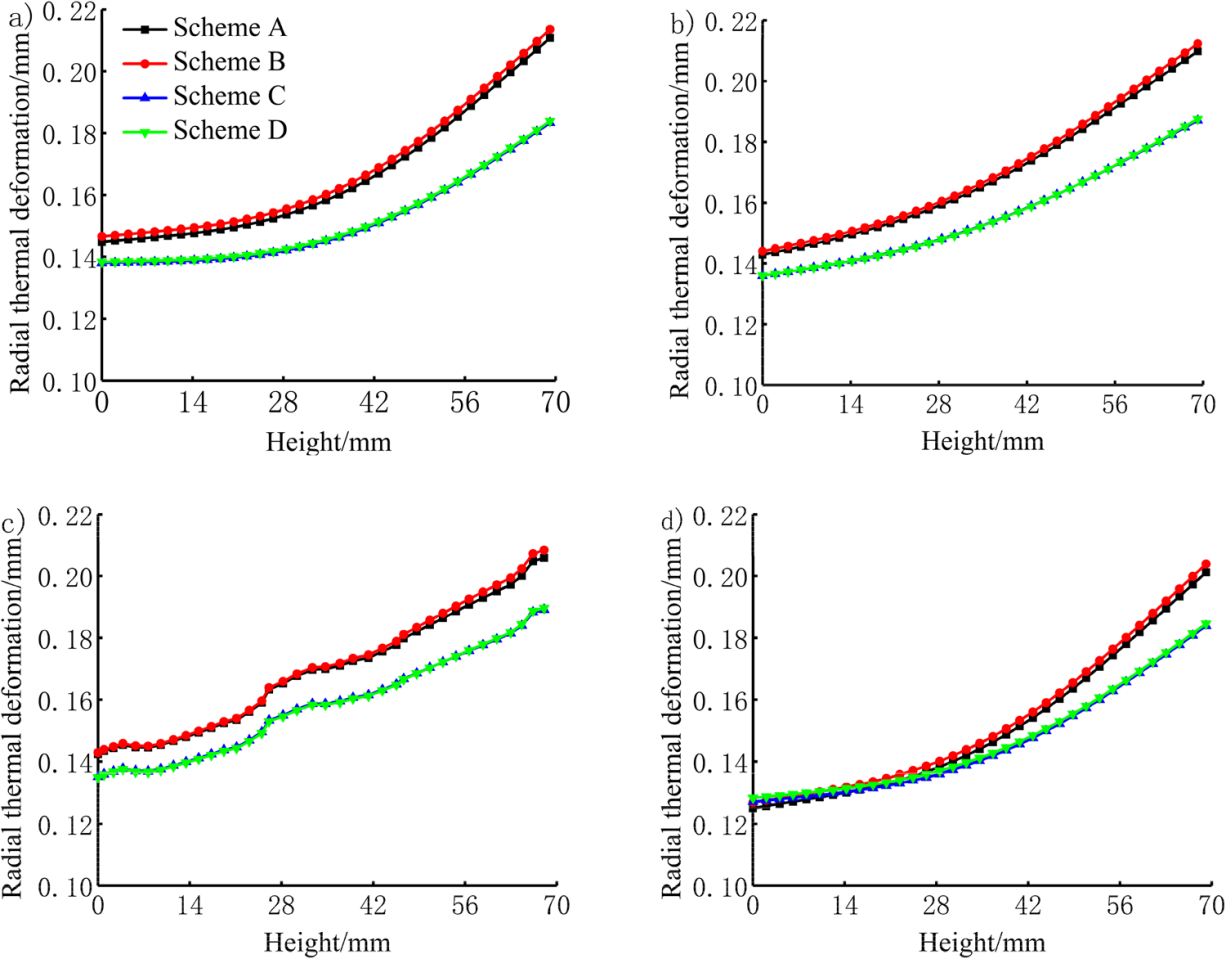
Radial thermal deformation in the axial direction of (**a**) 0° of the piston skirt, (**b**) 30° of the piston skirt, (**c**) 60° of the piston skirt and (**d**) 180° of the piston skirt.

The characteristic lines of the piston skirt in the directions of 0°, 30°, 60°, and 180° were extracted to study the radial thermal deformation of the piston in the axial direction. The bottom of the piston was at a height of 0 mm, as shown in Fig. 12. As seen in the figure, the radial thermal deformation at the top of the piston skirt is the largest and the deformation at the bottom is the smallest. The thermal deformations of the TBC piston and aluminium alloy piston under the maximum torque condition were slightly larger than those under the rated power condition. In the 0° direction, the maximum radial thermal deformation of the TBC piston under the rated power and maximum torque conditions were 0.183 mm and 0.184 mm, respectively, 13.3% and 14.0% lower than the deformations of 0.211 mm and 0.214 mm, respectively, similarly observed for the aluminium alloy piston. In the direction of 30°, the maximum radial thermal deformation of the TBC piston under the rated power and maximum torque conditions were 0.187 mm and 0.188 mm, respectively, 11.0% and 11.3% lower than the 0.210 mm and 0.212 mm deformations, respectively, of the aluminium alloy piston. In the 60° direction, the maximum radial thermal deformation of the TBC piston under the rated power and maximum torque conditions were 0.189 mm and 0.190 mm, respectively, 8.3% and 8.7% lower than the 0.206 mm and 0.208 mm deformations, respectively, of the aluminium alloy piston. In the window area, the radial thermal deformation of the piston fluctuated slightly owing to the change in the piston structure. In the 180° direction, the maximum radial thermal deformation of the TBC piston under the rated power and maximum torque conditions were 0.184 mm and 0.185 mm, 8.5% and 9.3% lower than the 0.201 mm and 0.204 mm deformations of the aluminium alloy piston.

**Figure 13:**
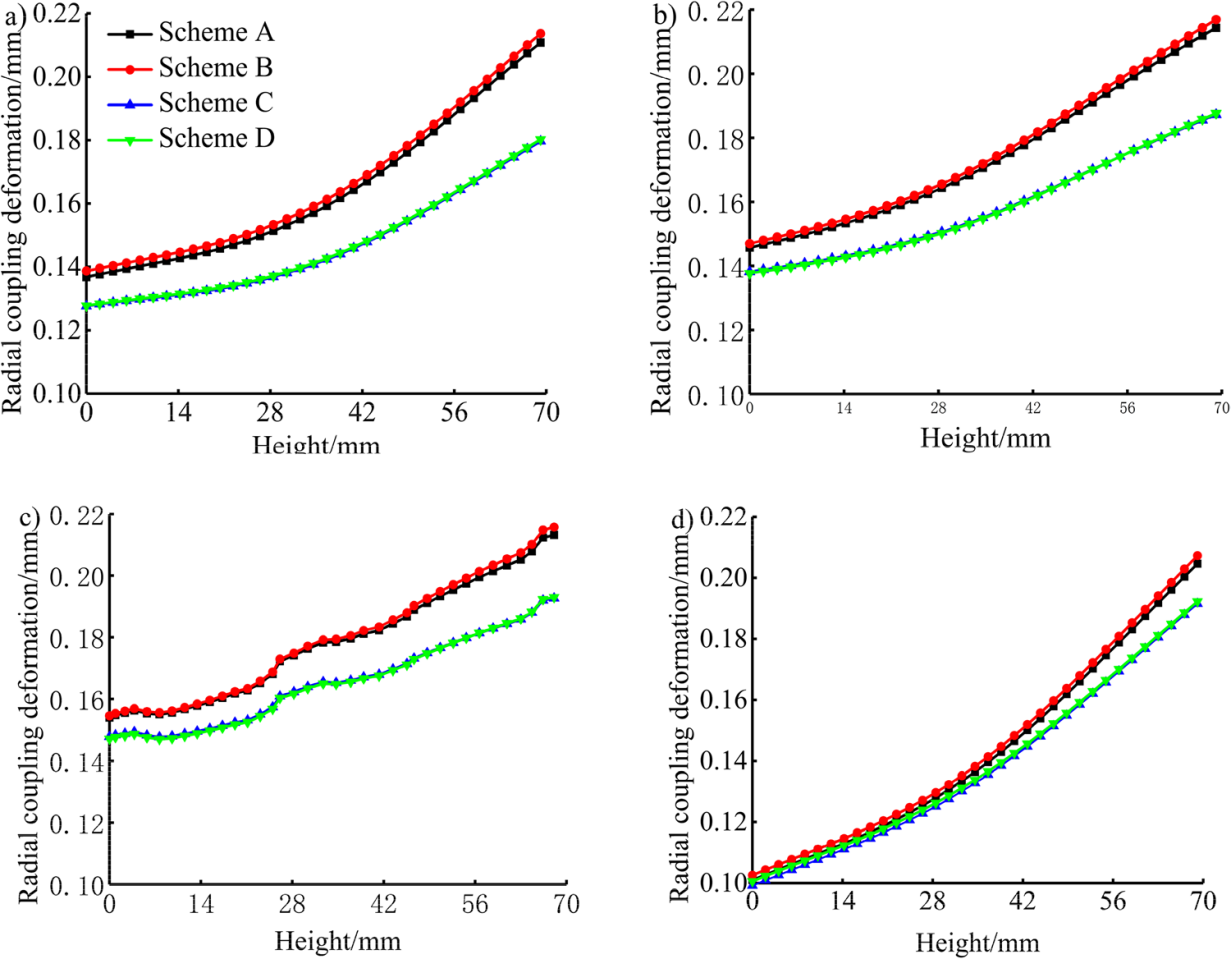
Radial coupling deformation in the axial direction of (**a**) 0° of the piston skirt, (**b**) 30° of the piston skirt, (**c**) 60° of the piston skirt and (**d**) 180° of the piston skirt.

The characteristic lines in the directions of 0°, 30°, 60°, and 180°of the piston skirtwere extracted to study the radial thermo-mechanical coupling deformation of the piston in the axial direction. The bottom of the piston was at a height of 0 mm, as shown in Fig. 13. As seen in the figure, the thermo-mechanical coupling deformation law of the piston skirt in each scheme is consistent with the thermal deformation. In the 0° direction, the maximum radial thermo-mechanical coupling deformation of the TBC piston under the rated power and maximum torque conditions were 0.180 mm and 0.180 mm, 14.7% and 15.9% lower than the 0.211 mm and 0.214 mm deformations, respectively, of the aluminium alloy piston. In the direction of 30°, the maximum radial thermo-mechanical coupling deformation of the TBC piston under the n rated power and maximum torque conditions were 0.187 mm and 0.188 mm, 12.6% and 13.4% lower than the 0.214 mm and 0.217 mm deformations, respectively, of the aluminium alloy piston. In the 60° direction, the maximum radial thermo-mechanical coupling deformation of the TBC piston under the rated power and maximum torque conditions were 0.192 mm and 0.193 mm, 9.9% and 10.6% respectively lower than the 0.213 mm and 0.216 mm deformations, respectively, of the aluminium alloy piston. In the 180° direction, the maximum radial thermo-mechanical coupling deformation of the TBC piston under the rated power and maximum torque conditions were 0.192 mm and 0.192 mm, 6.3% and 7.2% lower than the 0.205 mm and 0.207 mm deformations, respectively, of the aluminium alloy piston.

## Conclusions

In this study, the thermocouple measurement method was used to measure the steady-state temperature field of the aluminium alloy piston and the TBC piston under the rated power and maximum torque conditions. The temperature field provides accurate boundary conditions for the finite element analysis of the piston. Under this boundary condition, the temperature field, thermal stress, thermo-mechanical coupling stress, thermal deformation, and thermo-mechanical coupling deformation of aluminium alloy piston and TBC piston were systematically studied. Considering these factors, the following conclusions were drawn.

1. Using TBC results in the maximum temperature of the TBC piston decreasing by 12.2% and 13.73% compared with that of aluminium alloy piston under the rated power and maximum torque conditions, respectively, indicating that the TBC effectively prevents the heat transfer from the combustion chamber to the piston headand significantly reduces the temperature of the piston head.
2. The thermal insulation function of the ceramic layer reduces the temperature gradient of the piston, making the thermal stress of the TBC piston significantly lower than that of the aluminium alloy piston. Under the rated power and maximum torque conditions, the TBC piston exhibited a maximum decrease of 25.9% and 26.8%, respectively, at the top of the inner cavity compared with the aluminium alloy piston. Due to the restraining effect of the thermal barrier coating on its top surface, the TBC piston generates slightly higher thermo-mechanical coupling stresses—1.2 MPa and 3.7 MPa—at the bottom surface of the combustion chamber than the aluminium alloy piston under the rated power and maximum torque conditions, respectively.
3. The ceramic layer was closely combined with the top surface of the piston matrix, limiting the deformation of the top layer. The radial thermal deformation of the TBC piston under the rated power and maximum torque conditions was reduced by 0.067 mm and 0.073 mm, respectively, compared with that of the aluminium alloy piston; the radial thermo-mechanical coupling deformation was reduced by 0.069 mm and 0.075 mm, respectively. The radial thermal deformation of the piston in the direction parallel to the pinhole axis is greater than that in the perpendicular direction, with the difference in the magnitude resulting in the uneven thermal deformation of the piston.
